# Assessment of primary health care in a rural health centre in Enugu South east Nigeria

**DOI:** 10.12669/pjms.311.6360

**Published:** 2015

**Authors:** Josephat M Chinawa, Awoere T Chinawa

**Affiliations:** 1 Dr. Josephat M Chinawa, MBBS, FMCPaed, Lecturer, College of Medicine, Department of Pediatrics, University of Nigeria, University of Nigeria Teaching Hospital (UNTH), Ituku- Ozalla, Enugu State, Nigeria.; 2 Dr. Awoere T Chinawa, MBBS, FMCPH, Consultant Community Physician, University of Nigeria Teaching Hospital (UNTH), Ituku- Ozalla, Enugu State, Nigeria.

**Keywords:** Health centre, Immunization coverage, Children

## Abstract

**Objective:**

Primary health care (PHC) is a vital in any community. Any health centre with a well implemented PHC program can stand the test of time in curbing under five mortality and morbidity. This study was therefore aimed at assessing the activities in a health centre located in a rural area in Enugu state and to determine the pattern and presentation of various diseases in the health centre.

**Methods:**

This is retrospective study undertaken in a primary health care centre in Abakpa Nike in Enugu east LGA of Enugu State of Nigeria from December 2011 to December 31^st^ 2013. Data retrieved were collected with the aid of a structured study proforma and analyzed using SPSS Version 18.

**Results:**

Total number of children that attended immunization program in the health centre over 20 months period was 25,438 (12,348 males and 13090 females), however only 17745 children (7998 males and 9747 females) were actually registered in the hospital records. None of the children was immunized for DPT2 and OPV^0^ and HBV^1^ in the course of this study. The dropout rate using DPT1, 2 and 3 (DPT1-DPT2/DPT3) was very high (494%). The mean immunization coverage rate was 8.3%. Family planning activities, integrated management of childhood illnesses program were also carried out in the health centre but at very low level.

**Conclusions:**

The major fulcrum of events in the health centre which include immunization coverage, IMCI, and management of common illnesses were simply non operational. However the health centre had a well knitted referral system.

## INTRODUCTION

There is an immediate need to improve and provide quality health care among children in rural areas and suburban. This is necessary so as to avert the number of death that comes from that part of the country. Various health programs have been instituted in Enugu state aimed at providing and carrying out health care services in remote communities in Enugu and Ebonyi states where PHC operates.^1^

Many countries in the world have also taken several steps to strength the surveillance system especially for non-communicable diseases which have slowly and steadily been spreading their tentacles from the developed to the developing countries. For the health system of a developing country to thrive, the activities of her health centre powered by Primary health care should not be overlooked.^2^^,^^3^

Primary Health Care was defined by the World Health Organization in 1978 as essential health care; based on practical, scientifically sound, and socially acceptable method and technology; universally accessible to all in the community through their full participation; at an affordable cost; and geared toward self-reliance and self-determination.^4^ Primary health care is based on the overlap of mutuality, social justice and equality. As a strategy, primary health care focuses on individual and community strengths (assets) and opportunities for change; maximizes the involvement of the community; includes all relevant sectors but avoids duplication of services; and uses only health technologies that are accessible, acceptable, affordable and appropriate.^4^ Indeed, Primary health care need to be delivered close to the people. It is obvious that the purpose of PHC in the country is defeated since the government puts more emphasis on secondary and tertiary health care. This could be the reason why we have high burden of infant and maternal deaths.^5^

Poor and inappropriate management of our primary health care system and health centre in particular has contributed immensely to childhood morbidity and mortality. For instance, in 2001, 19 percent of global deaths were among children and 99 percent of all child deaths took place in low- and middle-income countries. The disability-adjusted life years (DALYs) lost was attributed to the under fives, maternal and perinatal conditions, nutrition deficiencies, and endocrine disorders.^6^^,^^7^

This study was therefore aimed at evaluating the activities of a health centre in a suburban in Enugu state and to determine the pattern and presentation of various diseases in the health centre. The findings from this study may help to improve management of children in health centers. In addition, it would also form a data base upon which further studies can be carried out.

## METHODS

This is retrospective study undertaken in a primary health care centre in Abakpa Nike in Enugu east LGA of Enugu State. The health Centre serves as a major primary health care for patients from Smaller villages such as ugwuago, ugboonye, ugbone,ugbowa etc. Abakpa Nike primary health centre was newly renovated by the Chairman of Enugu east LGA and provides services on immunization, integrated management of child hood illnesses, common and uncomplicated deliveries, and rarely management of non communicable disease.

Data on the activities of this health centre was collected from December, 2011 to 31^st^ December, 2013). This was extracted from the admissions register. Data extracted from the case record files included: age on admission, gender, Immunization pattern, IMCI procedures, family planning techniques, disease surveillance, diarrhea illnesses and management of non -communicable disease.

This study was aimed at assessing the activities in a health centre located in a rural region in Enugu state.

Ethical clearance for the study was sought from the chief nursing officer of the health centre. The data retrieved were collected with the aid of a structured study proforma and entered into a personal computer and analyzed with SPSS Version 18. Data were arranged in tables.

## RESULTS

Total number of children that attended immunization program in the health centre over 20 months period was 25,438 (12,348 males and 13090 females), however only 17745 children (7998 males and 9747 females) were actually registered in the hospital records. Table-I.

None of the children was immunized for DPT2 and OPV^0^ and HBV^1^ in the course of this study. Table-II. The dropout rate using DPT1, 2 and 3 (DPT1-DPT2/DPT3) was very high (494%). Immunization coverage rates for BCG, OPV^o^, OPV^1^_, _DPT^1^, HBV^0^, OPV^3^, HBV^3^, DPT^2^, DPT^3^, HBV^1^, measles and yellow fever were 11.7%, 0%, 15.3%, 16.4%, 4.1%, 13.6%, 14.3%, 0%, 3.3%, 0%, 11% and 10.3% respectively with mean coverage rate of 8.3%.

Family planning activities were poor, showing oral pills as mostly used family planning technique 107(79.3%), followed by condoms 11(8.1%),IUCD 10(7.4%) and least is injectables (5.2%). Table-III.

Two thousand, three hundred and eleven (2311) malaria cases were managed in the health centre over a 20 month period with 1172 males and 1139 females giving a male female ratio of 1:1.1. Out of these cases, 99 of them were severe malaria which were all referred. Table-IV.

Integrated management of childhood illnesses program was also carried out in health centre but at very low level. A total of 147 children benefited from this simple case management of diarrhea, cold, cough and fever.

Twenty one cases that were managed in the health centre were non communicable disease with hypertension 9(43%) being the commonest. Disease surveillance in this health centre was very poor as only cases of dysentery and diarrheal disease were being surveyed.

## DISCUSSION

This study had shown the level and importance of primary health care in this environment. Children under the age of five are in the majority that attended the primary health activities in this study. This includes immunization, management of common child hood disease such as malaria and integrated management of child hood illnesses. The reason for this high number of under fives in the health centre is obvious. This is because the mortality and morbidity of diseases in Nigeria is commonly seen among the under fives. For instance, one in every five children dies before their 5th birthday; most of these deaths can be prevented by immunization.^1^^,^^2^ In Nigeria, significant effort has been made in the past two decades towards the reduction of childhood morbidity and mortality through introduction of policies like improved immunization coverage, provision of good health facilities and increase in the number of health personnel.^8^^-^^11^ Despite these efforts, childhood mortality rate is still high. Recent data report infant and under-5 mortality rates of 88 and 14 deaths per 1000 live births respectively.^8^^-^^11^

From this study, it is also pertinent to note that more female children attended all the services in the health centre when compared to males. This is gratifying as parents are beginning to understand the importance of girl child and the need to implement the first “F” component of child survival strategy.

We noted with dissatisfaction the low immunization coverage rates in the health centre in view. Immunization coverage rates for BCG, OPV^o^, OPV^1 ^_,_DPT1 ,HBV^0^,OPV^3^, HBV^3^, DPT2, DPT3, HBV1, measles and yellow fever are 11.7%, 0%, 15.3%, 16.4%, 4.1%, 13.6%, 14.3%, 0%, 3.3%, 0%, 11% and 10.3% respectively with a mean coverage rate of 8.3%.This is lower than the mean coverage rate of 69.3% in West java and 43.5% obtained in India.^12^^,^^13^ However our result is even similar to the report of national coverage in Nigeria for full immunization, which is less than 13%, one of the lowest rates in the world. The reason for this lower coverage rates is mainly due to the insurgency in the northern part of the country .For instance, some states in northern Nigeria have coverage rates below 1%, and the average for the whole North West Zone is just 4%.^14^ Other reasons that can be offered for this low coverage rate in this study is non availability of some vaccines like OPV^0^, DPT^2^ and OPV^3^ in the time of study.

We also noted a very high dropout rate of 494% (assessed by the number of DPT1 and DPT3 that was given to the child over a year) in this study. This is due to unavailability of DPT^2^ in the course of study. We could not offer any reason for the unavailability of the vaccine. In 1990, reported three doses of diphtheria-pertussis-tetanus (DPT3) coverage in infants (<12 months of age) reached an estimated 56%.^15^ During the years following the global Universal Childhood Immunization efforts that culminated in 1990, immunization coverage rates in Nigeria declined significantly. Preliminary results of a 2006 national coverage survey reported 36% DPT3 coverage.^15^

Management of uncomplicated malaria and integrated management of child hood illnesses (IMCI) activities were seen in the health centre within the study period, but this is at lower level. The health centre also recorded no management of severe malaria as such cases are referred to secondary health centre. Integrated management of child hood illnesses (IMCI) is a strategy developed by the World Health Organizations’ Division of Child Health and Development and UNICEF. It has been introduced in more than 30 countries around the world to address morbidity and mortality in children under five years. The strategy focuses on the child in its whole entirety, rather than on a single disease or condition. Children often arrive at primary health care facilities with a number of diseases and have to be managed in an integrated manner at home and at the clinic. This strategy (IMCI) has helped to curb childhood mortality and mortality to a greater extent.

Non communicable diseases seen in the health centre were very few. This could be due to lack of trained professionals and facilities in the health centre. Most of the communicable diseases are often referred. This health centre has a very good referral system. For instance most of severe illnesses and complicated pregnancies are often referred to higher and well equipped government hospitals. This could account for the zero mortality rate that was documented in the hospital. This is surprising, because In Nigeria health centers, referral system can be said to be non-operational and this contributes especially to increased maternal and child morbidities and mortalities. There is no proper connection between the PHC and the secondary health facilities and in turn with the tertiary. Most major primary health centers in sub-Saharan Africa especially Nigeria provides primary and preventive services, making them overlabored.^16^^,^^17^ Disease surveillance, notification and documentation are almost non operational in the health centre under study.^18^ Surveillance and notification of disease is very important for the reporting of modifiable diseases.^18^ In Nigeria, the collection, collation, analysis, and interpretation of data in health-care facilities are often unsatisfactory, partly due to insufficient awareness among health-care personnel.^18^

**Table-I:** Age and gender of children attending the health center

	**M**	**F**
0-28 days	1101	1001
29 days -11 months- 12 months-	5753	6723
12 - 59 months	630	603
5 years - 14 years	277	150
>15 years	237	1270
Total	7998	9747

**Table-II:** Immunization coverage

	**M**	**F**
BCG	1331	1635
OPV^1^	1925	1956
DPT^1^	2043	2138
HB^0^	521	514
OPV^3^	1621	1831
HB^3^	1625	2001
DPT^3^	500	346
Measles	1504	1306
Yellow fever	1278	1363
Total	12,348	13090

**Table-III:** Frequency distribution of use of contraceptive Used

	**F**	**M**	**Total (%)**
Oral pills	107	0	107(79.3)
Injectables	7	0	7(5.2)
IUCD	10	0	10(7.4)
Condoms	0	11	11(8.1)

**Table-IV:** Frequencies of malaria cases that presented in the health centre

	**M**	**F**
< 5 years(UNM)	747	628
>= 5 years (UNM)	392	445
< 5years(Severe)	33(referred)	66(referred)
Total	1172	113(referred)

We noted much discrepancy between the numbers of children that attended immunization program in the health centre over 20 months period (25,438) and the number of children that were actually registered (17745) in the hospital records. This simply showed that the health centre in view had a poor recording system. Medical Record keeping had been a disturbing issue in some hospitals in Nigeria. For instance Afolabi^19^ noted that record management practice in Nigeria has a number of problems which may include insufficient skilled and experienced record management personnel and possibly, low priority of record management in the scheme of things. This was also corroborated by Awe.^20^ Hospitals are information intensive enterprises; hospital managers must understand that only those with a strong information management system can have a smooth running of the enterprise.^21^ In health care organizations, medical record is the principal repository of a patient's health care information, so every health organization needs a medical records department that is organized and staffed to provide adequate information.^21^

## CONCLUSION

The major fulcrum of events in the health centre which include immunization coverage, IMCI, and management of common illnesses are simply non operational. This is shown by the low coverage rate, high dropout rates, poor IMCI implementation and poor disease surveillance system. However the health centre had a well knitted referral system.

## Notes

***Funding Statement: ***This work is self sponsored; we bore all the expenses that accrued from this study.

## References

[1] Osa-Eloka CE, Nwakoby BA, Onajole AT, Chika NO, Nwobi EA (2009). Enhancing data management skills of primary health care workers in enugu state, Nigeria. Niger Postgrad Med J.

[2] Barcelon MA, Hardon A, Streefland P, Chabot J (1990). The community based health care program of the rural missionaries of the Philippines. Implementing primary health care.

[3] Cesar JA, Goncalves TS, Neumann NA, Oliveira Filho JA, Diziekaniak AC (2005). Child health in poor areas of North and Northeast Brazil: a comparison of areas covered by the Children's Mission and control areas. Cad Saude Publica.

[4] WHO Health Systems Strengthening Glossary – World.

[5] Murray CJL, Lopez AD, Mathers CD, Stein C (2001). The Global Burden of Disease 2000 project: aims methods and data sources.

[6] Arnesen TM, Norheim OF Disability Adjusted Life Years - possibilities and problems.

[7] World Health Organization About the Global Burden of Disease (GBD) project.

[8] UNICEF (2012). The State of the World’s Children.

[9] WHO and UNICEF UNICEF. Countdown to 2015. Building a Future for Women and Children - The 2012 Report.

[10] Nigeria (2008). Demographic and Health Survey.

[11] Black RE, Morris SS, Bryce J (2003). Where and why are 10 million children dying every year?. Lancet.

[12] Using Immunization Coverage Rates for Monitoring Health Sector Performance.

[13] (2005). Guidelines for Reporting and Management of Adverse Events Following Immunization.

[14] The State of Routine Immunization Services in Nigeria and Reasons for Current Problems.

[15] Keja K, Chan C, Hayden G, Henderson RH (1988). Expanded program on immunization. World Health Statistics.

[16] Akande TM (2004). Referral system in Nigeria: Study of a tertiary health facility. Ann Afr Med.

[17] Health Reform Foundation of Nigeria ( 2006). Child survival in Nigeria. Nigeria health review 2006.

[18] Chinomnso CN, Chika NO, Prosper OU, Ugochukwu UO (2012). Awareness and knowledge of disease surveillance and notification by health-care workers and availability of facility records in Anambra state, Nigeria. Niger Med J.

[19] Afolabi M (1999). Educational training archivists and record managers in Africa: annual conference procedure of the Society of Nigerian Archivists.

[20] Awe FA (2000). Principles and practice of schools record management: Paper presented at a records management course in Lagos State Public Service.

[21] Health inform antics (2006). Medical records management.

